# Meta-Analysis of Short-Term Outcomes After Robotic Pancreaticoduodenectomy in Octogenarians

**DOI:** 10.3390/geriatrics11010019

**Published:** 2026-02-13

**Authors:** Ahmed Hassan, Martyn Charles Stott, Sarthak Jain, Vasileios Kotsarinis, Hadiyat A. Ogunlayi, Lydia Loutzidou, Dimitrios Vouros, Amr Ebrahim, Shahin Hajibandeh, Shahab Hajibandeh, Jacob Kadamapuzha, Thomas Satyadas

**Affiliations:** 1 Department of Hepatobiliary and Pancreatic Surgery, Manchester Royal Infirmary Hospital, Manchester M13 9WL, UK; ahmed.hassan6@mft.nhs.uk (A.H.); martyncharles.stott@mft.nhs.uk (M.C.S.); sarthak.jain@mft.nhs.uk (S.J.); vasileios.kotsarinis@mft.nhs.uk (V.K.); hadiyat.ogunlayi@mft.nhs.uk (H.A.O.); lydia.loutzidou@mft.nhs.uk (L.L.); dimitrios.vouros@mft.nhs.uk (D.V.); amr.ebrahim@mft.nhs.uk (A.E.); jacob.kadamapuzha@mft.nhs.uk (J.K.); thomas.satyadas@mft.nhs.uk (T.S.); 2 Department of Hepatobiliary and Pancreatic Surgery, Queen Elizabeth Hospital, Birmingham B15 2GW, UK; shahin_hajibandeh@yahoo.com; 3 Faculty of Medicine, Health and Life Science, Swansea University, Swansea SA2 8PP, UK

**Keywords:** robotic, pancreaticoduodenectomy, octogenarian

## Abstract

**Background/Objectives:** To evaluate short-term postoperative outcomes in octogenarians undergoing robotic pancreaticoduodenectomy. **Methods:** In compliance with the PRISMA statement standards, a systematic review and random-effects meta-analysis was conducted. All studies reporting short-term postoperative outcomes in patients aged ≥ 80 undergoing robotic pancreaticoduodenectomy were included and analyzed. **Results**: A total of 321 octogenarians from five studies were included. The mean operative time was 459.7 min (95% CI 398.6–520.8) and the estimated intraoperative blood loss was 216.1 mL (95% CI 147.4–284.8). Conversion to open occurred in 3.8% (95% CI 0.0–7.7). The risk of postoperative mortality was 4.5% (95% CI 1.7–7.2) and Clavien-Dindo grade ≥ III (major) complications occurred in 28.0% (95% CI 22.9–33.1). The risk of grade B or C postoperative pancreatic fistula was 10% (95% CI 6.5–13.5). The hospital stay was 14.9 days (95% CI 10.2–19.5). The risk of reoperation and readmission were 8.0% (95% CI 4.4–11.7) and 25.6% (95% CI 16.9–34.3), respectively. Compared to patients aged <80, the risk of major complications was higher (OR: 1.81, *p* = 0.010) and hospital stay was longer (MD: 5.19 days, *p* = 0.030) in octogenarians. Compared to the open approach, robotic approach was associated with longer operative time (MD: 137.08 min, *p* = 0.0009), less intraoperative blood loss (MD: −246.00 mL, *p* = 0.010), and lower major complications (OR: 0.62, *p* = 0.020). **Conclusions**: Subject to selection and confounding bias, robotic pancreaticoduodenectomy may be safe with acceptable postoperative mortality and morbidity in highly selected octogenarians with good performance status. The results of the current study can be used for hypothesis synthesis and power analysis in future comparative studies.

## 1. Introduction

Due to the aging population, the number of people aged over 80 is estimated to reach 426 million by 2050 [1]. On the other hand, the incidence of pancreatic cancer peaks above the age of 80 [2]. Consequently, it is not uncommon to encounter an octogenarian with pancreatic cancer. Surgical resection remains the only potentially curative treatment for pancreatic cancer. While patients aged 80 years or over are conventionally less likely to be considered for surgical resection, modern medicine supports the argument that age alone should not be considered as a contraindication for surgical resection of pancreatic cancer [3].

Pancreaticoduodenectomy is a complex operation with a relatively high risk of morbidity and even mortality [4]. The available evidence supports the feasibility and safety of pancreaticoduodenectomy in highly selected octogenarians [5]. The recent advances in technology and operative techniques have resulted in the implementation of robotic approach in hepato−pancreato−biliary surgery [6,7]. This has encouraged researchers to evaluate whether three-dimensional visualization, tremor stabilization, use of wristed instruments, and ergonomic superiority associated with a robotic approach would result in improved outcomes in octogenarians. The safety and feasibility of robotic pancreaticoduodenectomy in octogenarians have been evaluated in recent observational studies, supporting a rationale for conducting a systematic review. Consequently, we aimed to conduct a systematic review and meta-analysis to evaluate short-term postoperative outcomes in octogenarians undergoing robotic pancreaticoduodenectomy.

## 2. Materials and Methods

### 2.1. Compliance with Reporting and Methodological Standards

The Cochrane Handbook for Systematic Reviews (version 6.4) [8] was followed to design and conduct the current study. The Preferred Reporting Items for Systematic reviews and Meta-Analyses (PRISMA) 2020 statement standards [9] were followed to describe the rationale and objectives, to report the methods and results, and to discuss the findings and conclusions. The study followed a pre-defined protocol which was registered in PROSPERO, which is a publicly available international database of prospectively registered systematic reviews (PROSPERO registration number: CRD420261277490). There was no deviation from the registered protocol.

### 2.2. Eligibility Criteria for Study Selection

The eligible studies included in the current study met the following principal eligibility criteria in terms of study design, included population, interventions, and outcomes:

- Study design: All studies (randomised controlled trials, retrospective and prospective cohort studies, and case series) with a minimum sample size of 15 patients were eligible for inclusion. Meta-analyses, systematic reviews, review articles, case-control studies, case reports, letters, correspondences, and opinions were excluded.
- Population: All patients age ≥ 80 with an indication for pancreaticoduodenectomy were considered eligible for inclusion.
- Intervention: Robotic pancreaticoduodenectomy (pylorus preserving pancreaticoduodenectomy or Whipple procedure) was the intervention of interest.
- Outcomes: The outcomes of interest included operative time, intraoperative blood loss, conversion to open surgery, postoperative mortality, Clavien-Dindo grade ≥ III complications, grade B or C postoperative pancreatic fistula (POPF), length of hospital stay, reoperation, and readmission.

### 2.3. Search Strategy

Based on the eligibility criteria of the current study, a comprehensive search strategy with no language restrictions was developed by two authors with experience in evidence synthesis. The following databases were searched: MEDLINE, Scopus, CENTRAL, the ISRCTN registry, the ICTRP registry, and ClinicalTrials.gov. The search strategy included the following combination of keywords: (robot OR robotic) AND (pancreaticoduodenectomy [MeSH Terms] OR pancreatectomy [MeSH Terms] OR pancreaticoduodenectomy OR pancreatectomy OR whipples) AND (octogenarian [MeSH Terms] OR octogenarian OR 80 year [Title] OR 80 [Title]). In addition to the above, the reference lists of potentially eligible articles were screened to identify more eligible studies. The date of the most recent search was 1 December 2025.

### 2.4. Screening and Selection of the Eligible Articles

The title and abstract of the articles were found by searching the above databases were screened by two independent authors. The principal eligibility criteria were strictly respected; irrelevant articles were excluded based on titles and abstracts without full-text review. The full-text of potentially relevant articles were reviewed and those meeting the principal eligibility criteria were included. If there was any discrepancy between the findings of the two independent authors, it was resolved by discussion and involvement of third independent author.

### 2.5. Data Variables and Data Collection

The data variables were determined at the protocol development stage, which was then optimized after study selection using the pilot-testing technique. The data collection sheet included three categories of data variables: (1) variables related to bibliography and design of each study; (2) variables related to baseline characteristics of each study; and (3) variables related to the outcomes. Two independent authors collected the information related to the above variables. If there was any discrepancy between the findings of the two independent authors, it was resolved by discussion and involvement of third independent author. The following variables were included in the data collection sheet: first author, year of study, country of the study, journal of the article, study design of the study, sample size, age, sex, operative time, intraoperative blood loss, conversion to open surgery, postoperative mortality, Clavien-Dindo grade ≥ III complications, grade B or C POPF, length of hospital stay, reoperation, and readmission.

### 2.6. Study Risk of Bias and Evidence Certainty Assessment

The risk of bias in the included studies was evaluated using the Risk Of Bias In Non- Randomized Studies of Interventions (ROBINS-I) tool by two independent authors. The certainty of evidence was evaluated using the GRADE system. If there was any discrepancy between the findings of the two independent authors, it was resolved by discussion and involvement of third independent author.

### 2.7. Statistical Analyses

The statistical analyses were performed using OpenMeta[Analyst] software for single-arm meta-analysis and RevMan Web for comparison meta-analysis. In the single-arm meta-analysis, the random-effects model was used to calculate pooled outcomes with 95% confidence intervals (CIs). In the comparison of the meta-analysis model, the random-effects model was used to calculate the odds ratio (OR) for the dichotomous outcomes and the mean difference (MD) for the continuous outcomes. The unit of analysis was individual patient. The results were presented in forest plots with 95% confidence intervals (CIs). Statistical heterogeneity was quantified as I^2^ using Cochran’s Q test (χ^2^), and heterogeneity was classified as low when I^2^ was 0–25%, moderate when I^2^ was 25–75%, and high when I^2^ was 75–100%. The protocol planned to evaluate the risk of publication bias by visual assessment of the funnel plot if the outcomes were reported by a minimum of 10 studies; however, this was not done as the number of included studies was less than 10.

### 2.8. Sensitivity Analyses

Leave-one-out analysis was performed as part of the sensitivity analysis. Moreover, studies with low risk of bias were analysed separately.

## 3. Results

### 3.1. Search Results

**Figure 1:**
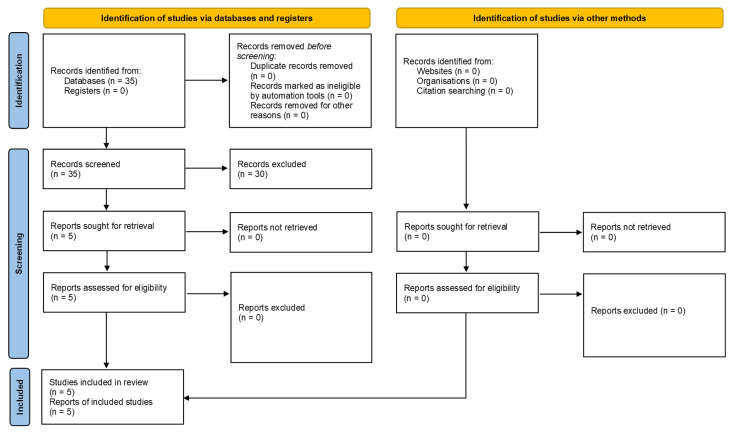
Study PRISMA flow diagram.

The study PRISMA flow diagram is shown in Figure 1. The search of databases resulted in 35 articles. Screening the titles and abstracts resulted in exclusion of 30 articles without full-text review due to being irrelevant to the principal eligibility criteria. The full-text review of the remaining five articles resulted inclusion of all them. Consequently, five studies [10,11,12,13,14] including 321 octogenarians were eligible for inclusion. Baseline characteristics of the included studies are shown in Table 1.

### 3.2. Study Risk of Bias Assessment

Based on the ROBINS-I risk of bias assessment tool, all studies were considered to be at high risk of bias in terms of confounding and participant selection domains. However, all studies were considered to be at low risk of bias in terms of intervention classification, deviation from intended interventions, missing data, outcome measurement, and selective reporting domains (Table 1).

### 3.3. Single-Arm Meta-Analysis

#### 3.3.1. Operative Time

**Figure 2:**
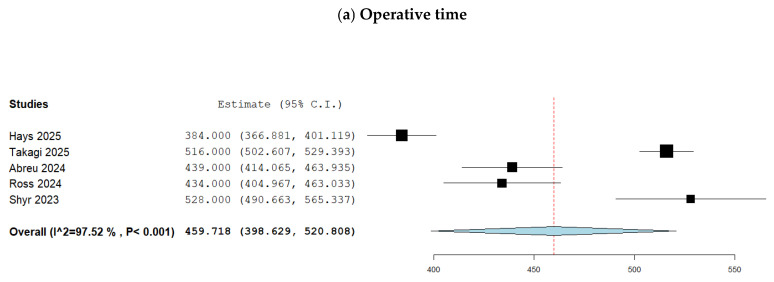

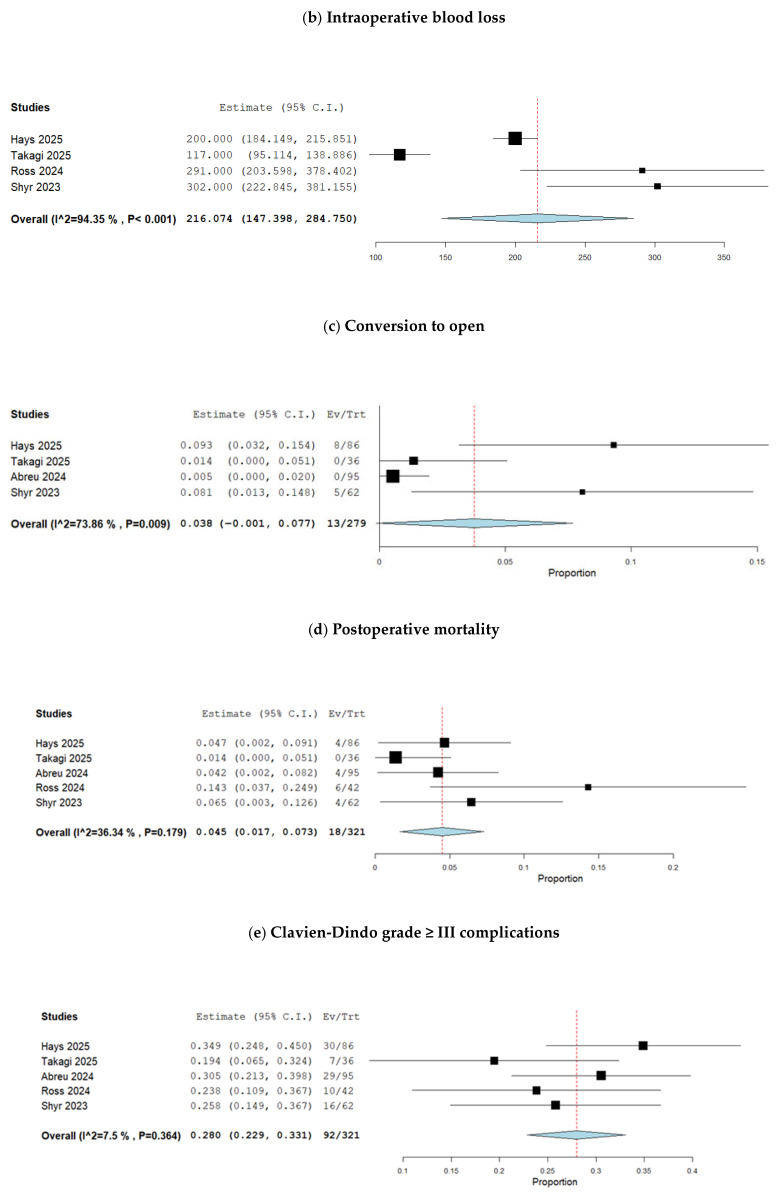

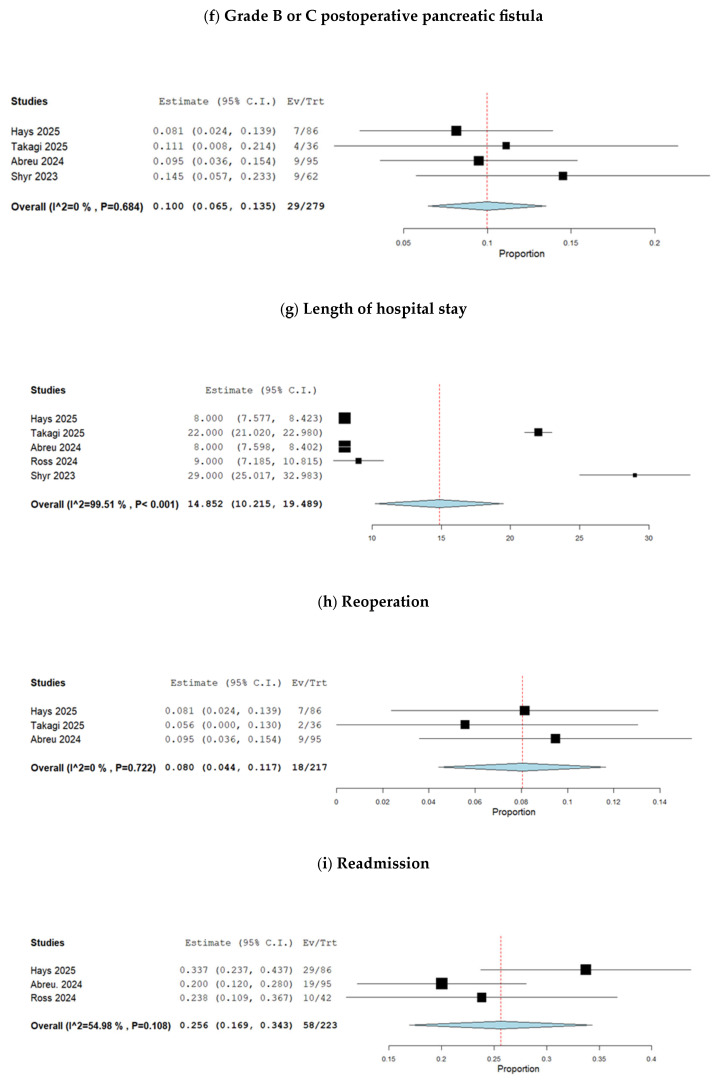
Forest plots for the outcomes after pancreaticoduodenectomy in octogenarians: (**a**) operative time; (**b**) intraoperative blood loss; (**c**) conversion to open; (**d**) postoperative mortality; (**e**) Clavien-Dindo grade ≥ III complications; (**f**) grade B or C postoperative pancreatic fistula; (**g**) length of hospital stay; (**h**) reoperation; (**i**) readmission. Hays et al. [10], Takagi et al. [11], Abreu et al. [12], Ross et al. [13], Shyr et al. [14].

Analysis of 321 patients from five studies showed that the mean operative time was 459.7 min (95% CI 398.6–520.8) (Figure 2a). The between-study statistical heterogeneity was high (I^2^ = 98%, *p* < 0.001) and the GRADE certainty was low (Supplementary Table S1).

#### 3.3.2. Intraoperative Blood Loss

Analysis of 226 patients from four studies showed that the estimated intraoperative blood loss was 216.1 mL (95% CI 147.4–284.8) (Figure 2b). The between-study statistical heterogeneity was high (I^2^ = 94%, *p* < 0.001) and the GRADE certainty was low (Supplementary Table S1).

#### 3.3.3. Conversion to Open

Analysis of 279 patients from four studies showed that the likelihood of conversion to open surgery was 3.8% (95% CI 0.0–7.7) (Figure 2c). The between-study statistical heterogeneity was moderate (I^2^ = 74%, *p* = 0.009) and the GRADE certainty was moderate (Supplementary Table S1).

#### 3.3.4. Postoperative Mortality

Analysis of 321 patients from five studies showed that the risk of postoperative mortality was 4.5% (95% CI 1.7–7.2) (Figure 2d). The between-study statistical heterogeneity was moderate (I^2^ = 36%, *p* = 0.179) and the GRADE certainty was moderate (Supplementary Table S1).

#### 3.3.5. Clavien-Dindo Grade ≥ III Complications

Analysis of 321 patients from five studies showed that Clavien-Dindo grade ≥ III complications occurred in 28.0% (95% CI 22.9–33.1) (Figure 2e). The between-study statistical heterogeneity was low (I^2^ = 8%, *p* = 0.364) and the GRADE certainty was moderate (Supplementary Table S1).

#### 3.3.6. Grade B or C POPF

Analysis of 279 patients from four studies showed that the risk of grade B or C POPF was 10% (95% CI 6.5–13.5) (Figure 2f). The between-study statistical heterogeneity was low (I^2^ = 0%, *p* = 0.684) and the GRADE certainty was moderate (Supplementary Table S1).

#### 3.3.7. Length of Hospital Stay

Analysis of 321 patients from five studies showed that the mean length of hospital stay was 14.9 days (95% CI 10.2–19.5) (Figure 2g). The between-study statistical heterogeneity was high (I^2^ = 99%, *p* < 0.001) and the GRADE certainty was low (Supplementary Table S1).

#### 3.3.8. Reoperation

Analysis of 217 patients from three studies showed that the risk of reoperation was 8.0% (95% CI 4.4–11.7) (Figure 2h). The between-study statistical heterogeneity was low (I^2^ = 0%, *p* = 0.722) and the GRADE certainty was moderate (Supplementary Table S1).

#### 3.3.9. Readmission

Analysis of 223 patients from three studies showed that the risk of readmission was 25.6% (95% CI 16.9–34.3) (Figure 2i). The between-study statistical heterogeneity was moderate (I^2^ = 55%, *p* = 0.108) and the GRADE certainty was moderate (Supplementary Table S1).

### 3.4. Comparison Meta-Analysis

#### 3.4.1. Age ≥ 80 Versus Age < 80

**Figure 3:**
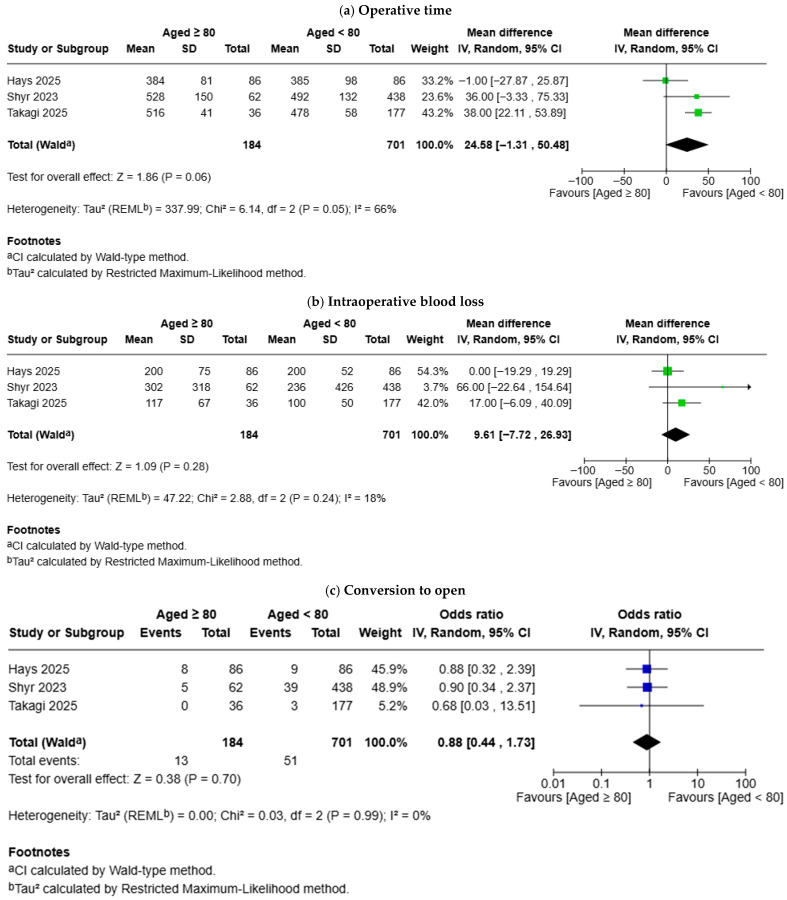

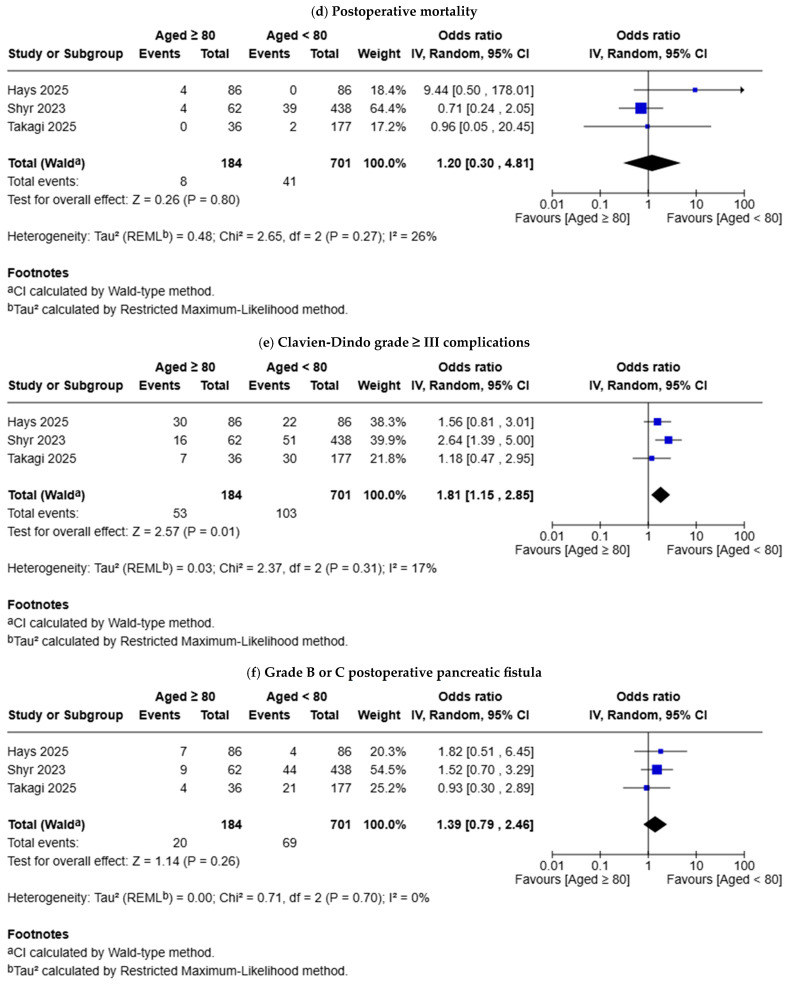

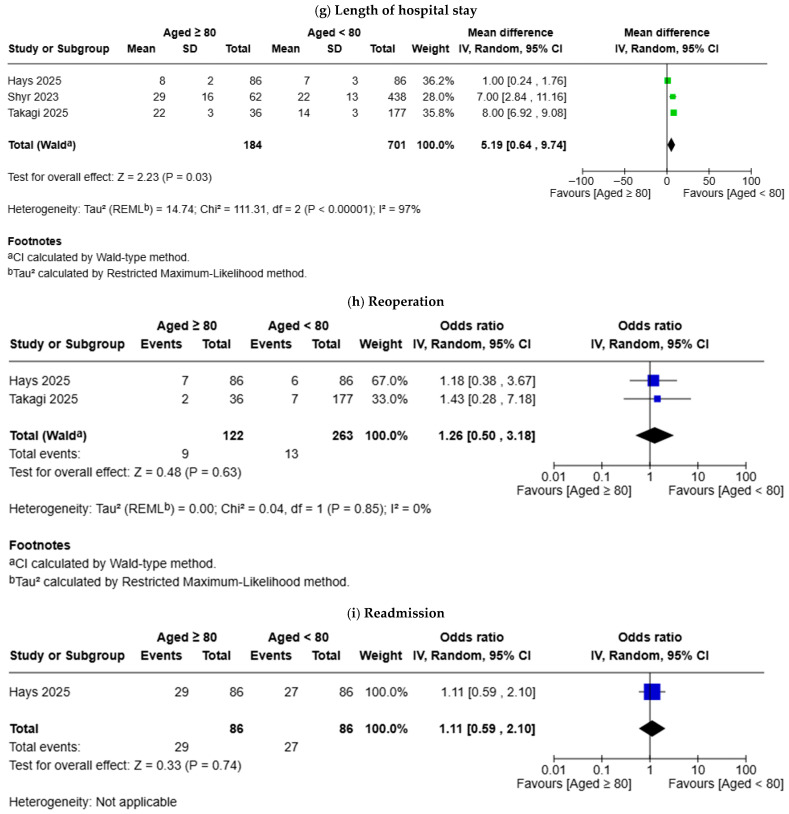
Forest plots for comparison of outcomes between octogenarians and patients aged < 80: (**a**) operative time; (**b**) intraoperative blood loss; (**c**) Conversion to open; (**d**) Postoperative mortality; (**e**) Clavien-Dindo grade ≥ III complications; (**f**) Grade B or C postoperative pancreatic fistula; (**g**) Length of hospital stay; (**h**) Reoperation; (**i**) Readmission. Hays et al. [10], Takagi et al. [11], Shyr et al. [14].

Three studies compared the outcomes between patients aged ≥ 80 and those aged < 80 (Figure 3). There was no difference in operative time (MD: 24.58 min, 95% CI −1.31–50.48, *p* = 0.060), intraoperative blood loss (MD: 9.61 mL, 95% CI −7.72–26.93, *p* = 0.280), conversion to open (OR: 0.88, 95% CI 0.44–1.73, *p* = 0.700), postoperative mortality (OR: 1.20, 95% CI 0.30–4.81, *p* = 0.800), grade B or C POPF (OR: 1.39, 95% CI 0.79–2.46, *p* = 0.260), reoperation (OR: 1.26, 95% CI 0.50–3.18, *p* = 0.630), and readmission (OR: 1.11, 95% CI 0.59–2.10, *p* = 0.740) between octogenarians and patients aged < 80. The risk of Clavien-Dindo grade ≥ III complications (OR: 1.81, 95% CI 1.15–2.85, *p* = 0.010) was higher in octogenarians and the length of hospital stay (MD: 5.19 days, 95% CI 0.64–9.74, *p* = 0.030) was longer in octogenarians.

#### 3.4.2. Robotic Approach Versus Open Approach

**Figure 4:**
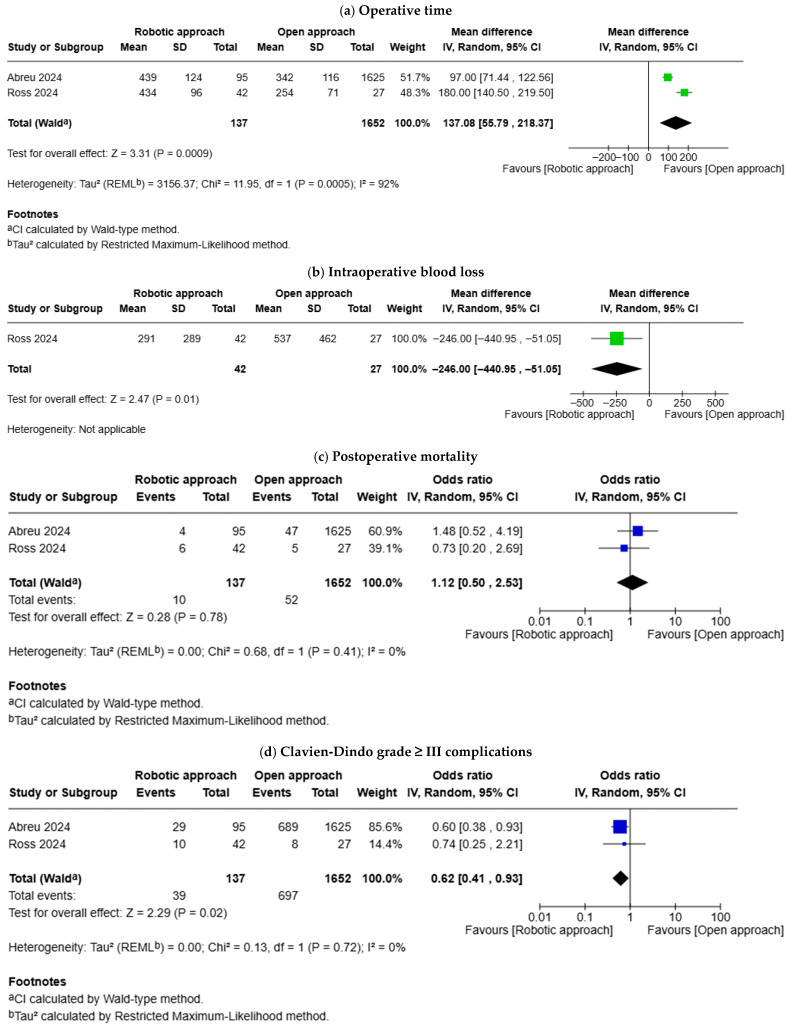

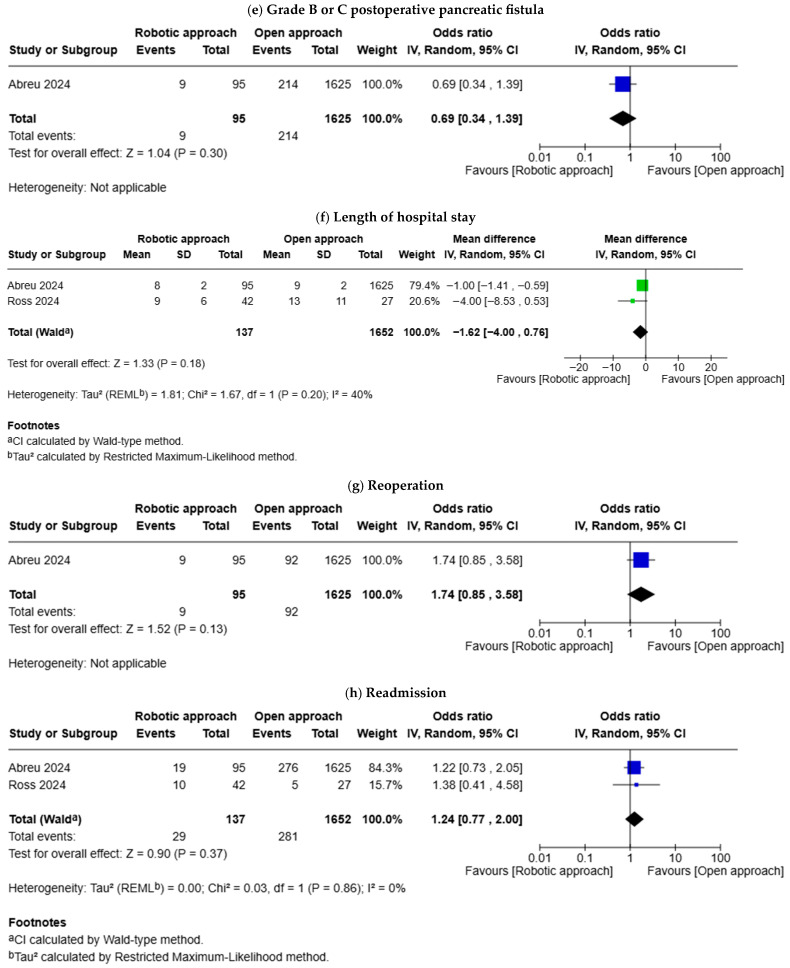
Forest plots for comparison of outcomes between octogenarians and patients aged < 80: (**a**) operative time; (**b**) intraoperative blood loss; (**c**) postoperative mortality; (**d**) Clavien-Dindo grade ≥ III complications; (**e**) Grade B or C postoperative pancreatic fistula; (**f**) length of hospital stay; (**g**) reoperation; (**h**) readmission. Abreu et al. [12], Ross et al. [13].

Two studies compared the outcomes between robotic approach and open approach (Figure 4). Robotic approach was associated with longer operative time (MD: 137.08 min, 95% CI 55.79–218.37, *p* = 0.0009), less intraoperative blood loss (MD: −246.00 mL, 95% CI −440.95–−51.05, *p* = 0.010), and lower risk of Clavien-Dindo grade ≥ III complications (OR: 0.62, 95% CI 0.41–0.93, *p* = 0.020). There was no difference in postoperative mortality (OR: 1.12, 95% CI 0.50–2.53, *p* = 0.780), grade B or C POPF (OR: 0.69, 95% CI 0.34–1.39, *p* = 0.300), length of hospital stay (MD: −1.62 days, 95% CI −4.00–0.76, *p* = 0.180), reoperation (OR: 1.74, 95% CI 0.85–3.58, *p* = 0.130), and readmission (OR: 1.24, 95% CI 0.77–2.00, *p* = 0.370) between the robotic approach and open approach.

### 3.5. Sensitivity Analysis

Leave-one-out analysis and separate analyses of studies with low risk of bias showed consistency of the results.

## 4. Discussion

A systematic review with meta-analysis was conducted to evaluate short-term postoperative outcomes in octogenarians undergoing robotic pancreaticoduodenectomy. Analysis of 321 octogenarians from five studies showed that robotic pancreaticoduodenectomy may be associated with safe and acceptable postoperative mortality and morbidity outcomes in highly selected octogenarians with a good performance status. Compared to patients aged <80, the risk of major complications was higher and hospital stay was longer in octogenarians. Compared to the open approach, the robotic approach was associated with longer operative time, less intraoperative blood loss, and lower major complications. The GRADE certainty of the available evidence was low to moderate.

The short-term postoperative outcomes after robotic pancreaticoduodenectomy in octogenarians has not been evaluated in previous systematic reviews; however, the results can be compared with the findings of systematic reviews in non-robotic settings. Kim et al. [4] conducted a meta-analysis of 18 studies and concluded that pancreaticoduodenectomy in octogenarians is associated with higher risk of morbidity and mortality compared to younger patients, hence the study recommended careful selection of octogenarians for pancreaticoduodenectomy [4]. This was consistent with the findings of a meta-analysis conducted by Phillipos et al. [15], concluding that elderly patients should undergo careful preoperative assessment and selection for consideration of pancreatic surgery [15]. In another study, Tan et al. [3] conducted a meta-analysis of 12 studies which concluded that pancreatic resection on elderly patients may be performed safely in experienced centres [3].

Based on the results of the current study, supported by findings of the aforementioned systematic reviews, it may be argued that in highly selected octogenarians robotic pancreaticoduodenectomy may be safe and feasible. This may highlight that objective measurement of age-related physiological decline and vulnerability, such as sarcopenia or clinical frailty score, may be better predictors of complications than age on its own [16,17]. Consequently, octogenarians with good performance status, appropriate physiological reserve, and muscle mass may be considered for pancreatic resection after objective assessment of their fitness for surgery.

The need for reoperation was 8.0% (95% CI 4.4–11.7) in the current study. Although the included studies did not report the reasons for reoperation, the risk of reoperation found in this study is consistent with the findings of other studies in younger patients. Lyu et al. [18] reported a reoperation rate of 5.9% after pancreaticoduodenectomy [18]. In another study, Qiu et al. [19] reported reoperation rate of 6.7% after pancreaticoduodenectomy [19]. Therefore, the reoperation rate found in this study can be justified by the results of previous studies.

It has been shown that robotic pancreaticoduodenectomy is a safe and feasible alternative to open pancreaticoduodenectomy in younger patients [20]. While the current study showed that the robotic approach was associated with a longer operative time, less intraoperative blood loss, and lower major complications compared with the open approach, the comparative evidence in octogenarians remains very limited. The robotic approach has advantages, including three-dimensional visualisation, tremor stabilization, use of wristed instruments, and ergonomic superiority, which allows for the completion of a major complex operation without a need for larger incisions. Whether these advantages translate into improved outcomes compared with open surgery should be investigated in future studies, and the results of current study can be used for hypothesis synthesis and power analysis in future comparative studies.

The current study has the following limitations. The included studies in this meta-analysis are subject to inevitable selection bias, confounding by indication, and confounding by fitness for operation due to their retrospective design. The included studies included patients who were already a fit candidate for pancreaticoduodenectomy and a large proportion of patients who were considered as high risk for pancreaticoduodenectomy were not included. Moreover, the between-study heterogeneity was high for operative time, intraoperative blood loss, and length of hospital stay. The observed heterogeneity can be explained by the variations among the included centres in terms of setting, and robotic surgery volume or experience. On the other hand, meta-analysis of continuous variables is usually associated with higher statistical heterogeneity compared with meta-analysis of dichotomous variables. Nevertheless, we downgraded the certainty of evidence to take into account the observed high statistical heterogeneity, and we performed sensitivity of analyses to evaluate robustness of the results. Finally, publication bias could not be assessed formally because the number of included studies was less than 10.

## 5. Conclusions

Subject to selection and confounding bias, robotic pancreaticoduodenectomy may be safe with acceptable postoperative mortality and morbidity in highly selected octogenarians with a good performance status. The results of current study can be used for hypothesis synthesis and power analysis in future comparative studies.

## Figures and Tables

**Table 1:** Baseline characteristics of the included studies.

Study, Year, Country	Journal	Design	Included Patients	Sample Size	ROBINS-I Risk of Bias Assessment						
					Confounding	Participants Selection	Intervention Classification	Deviations from Intended Interventions	Missing Data	Outcomes Measurement	Selective Reporting
Hays et al. [10], 2025, USA	HPB	Retrospective observational	Patients age ≥ 80 undergoing robotic pancreaticoduodenectomy	86	High risk of bias	High risk of bias	Low risk of bias	Low risk of bias	Low risk of bias	Low risk of bias	Low risk of bias
Takagi et al. [11], 2025, Japan	Cancers	Retrospective observational	Patients age ≥ 80 undergoing robotic pancreaticoduodenectomy	36	High risk of bias	High risk of bias	Low risk of bias	Low risk of bias	Low risk of bias	Low risk of bias	Low risk of bias
Abreu et al. [12], 2024, USA	HPB	Retrospective observational	Patients age ≥ 80 undergoing robotic pancreaticoduodenectomy	95	High risk of bias	High risk of bias	Low risk of bias	Low risk of bias	Low risk of bias	Low risk of bias	Low risk of bias
Ross et al. [13], 2024, USA	Journal of Robotic Surgery	Retrospective observational	Patients age ≥ 80 undergoing robotic pancreaticoduodenectomy	42	High risk of bias	High risk of bias	Low risk of bias	Low risk of bias	Low risk of bias	Low risk of bias	Low risk of bias
Shyr et al. [14], 2023, China	Clinical Interventions in Aging	Retrospective observational	Patients age ≥ 80 undergoing robotic pancreaticoduodenectomy	62	High risk of bias	High risk of bias	Low risk of bias	Low risk of bias	Low risk of bias	Low risk of bias	Low risk of bias

## Data Availability

The corresponding author (S.H. (Shahab Hajibandeh)) will provide any additional data related to this study upon reasonable request.

## Supplementary Materials

The following supporting information can be downloaded at: https://www.mdpi.com/article/10.3390/geriatrics11010019/s1, Table S1. Summary of findings table for single-arm meta-analysis model; Table S2. PRISMA 2020 Checklist.
